# Rogers Syndrome and Callosal Disconnection in the Setting of Moyamoya Disease

**DOI:** 10.7759/cureus.94194

**Published:** 2025-10-09

**Authors:** Carlos A Suanes Zubizarreta, Aorzala Atmar, Grayson V Gigliotti, Mina T Rizk

**Affiliations:** 1 Department of Translational Medicine, Florida International University, Herbert Wertheim College of Medicine, Miami, USA; 2 Neurology, Baptist Health Miami Neuroscience Institute, Baptist Health South Florida, Miami, USA

**Keywords:** callosal disconnection syndrome, moya moya disease, moyamoya disease (mmd), moyamoya treatment, roger’s syndrome, stroke, thiamine-responsive megaloblastic anemia (trma)

## Abstract

Rogers syndrome, also known as thiamine-responsive megaloblastic anemia (TRMA), is a rare autosomal recessive disorder characterized by diabetes mellitus (DM), sensorineural hearing loss, and megaloblastic anemia responsive to thiamine supplementation. Moyamoya disease is a progressive steno-occlusive cerebrovascular disorder involving stenosis of the terminal internal carotid arteries with subsequent formation of fragile collateral vessels, resulting in ischemic or hemorrhagic strokes. We present the first documented case of the co-occurrence of Rogers syndrome and moyamoya disease, involving a 22-year-old female who presented with altered mental status, left-sided weakness, and delayed speech. Neuroimaging revealed acute infarction of the corpus callosum and bilateral watershed territories, manifesting as callosal disconnection syndrome. The patient underwent combined direct and indirect revascularization procedures followed by multidisciplinary rehabilitative care. This report underscores the complex interplay of rare genetic, metabolic, and vascular factors leading to atypical stroke presentations and highlights the importance of thorough evaluation and coordinated multidisciplinary management in young patients with syndromic cerebrovascular disease.

## Introduction

Thiamine-responsive megaloblastic anemia (TRMA), also known as Rogers syndrome, is a rare autosomal recessive disorder characterized by diabetes mellitus (DM), sensorineural hearing loss, and megaloblastic anemia that responds to thiamine treatment [1]. Recent research has found gene mutations in the SLC19A2 gene that code for a plasma membrane thiamine carrier protein [2]. The exact prevalence of Rogers syndrome is unknown. However, there are only about 100 documented cases in medical literature [3]. Common presentations involve type 1 diabetes diagnosed in childhood and may be accompanied by developmental delays and unspecified neurological symptoms. The presentation also includes progressive sensorineural hearing loss and megaloblastic anemia that is nonresponsive to B12 and B9 treatment but responsive to B1 supplementation.

The diagnosis of Rogers syndrome is primarily clinical, in addition to confirmatory laboratory tests, beginning with the triad of type 1 diabetes, sensorineural hearing loss, and megaloblastic anemia. Genetic testing is then performed to identify mutations in the SLC19A2 gene for confirmation of the diagnosis. The current treatments include high-dose thiamine supplementation. In some cases, early supplementation of thiamine reduces the need for insulin management of type 1 diabetes [1]. Additionally, the management may include the management of type 1 diabetes with insulin and necessary hypoglycemic agents, hearing aid or cochlear implants for hearing loss, and supportive care to manage the overall spectrum of symptoms found in Rogers syndrome [1]. 

Moyamoya disease is a rare cerebrovascular occlusive disease characterized by unilateral or bilateral stenosis of the terminal portion of the internal carotid artery with subsequent formation of abnormal vascular networks at the base of the brain [4,5,6]. The abnormal vessels are termed moyamoya vessels, which come from the Japanese word for smoke (“moyamoya”) because they resemble a puff of smoke on cerebral angiography. The idiopathic form is termed moyamoya disease, whereas moyamoya syndrome is used to describe individuals who meet the criteria for moyamoya disease and have an associated comorbid medical condition [4,6]. In North America, the incidence of moyamoya is 0.1 per 100,000 individuals. While in East Asian countries, the annual incidence is 0.5-1.5 per 100,000. The age of onset is bimodal, with peaks occurring in the first decade of life and then again at around 40 years. The disease presents as an ischemic or hemorrhagic stroke of varying severity, seizures, and headache [4].

Conventional catheter cerebral angiography is the gold standard for the diagnosis of moyamoya disease [4] and demonstrates stenosis or occlusion in the arteries centered on the terminal portion of the intracranial internal carotid artery and abnormal vascular networks (moyamoya vessels) near the lesions [6]. Additionally, less invasive imaging can be used, such as CT perfusion, CT angiography, MRI, MR angiography (MRA), and MR perfusion [4]. However, the most recent diagnostic criteria from Japan emphasize the importance of cerebral angiography to differentiate moyamoya from atherosclerosis and other diseases [6]. Disease-specific drug treatment is not currently available [4]. Antiplatelet therapy has been suggested to improve survival, but randomized controlled trials are necessary to confirm the findings [7].

When the disease presents with cerebral ischemic symptoms, surgical revascularization is recommended, which may be direct, indirect, or a combination of both [4,8]. Revascularization surgery is also recommended for the management of hemorrhage to prevent recurrence. The most common areas of ischemic disease are the watershed areas of the anterior circulation. Very infrequently, strokes can affect the callosal fibers and lead to classic callosal disconnection syndrome, which is characterized by unilateral ideomotor apraxia, left-hand agraphia, left tactile anomia, left visual field alexia, intermanual conflict, and deficits in bimanual coordination. The extent of the disruption to the interhemispheric connection depends on the area and amount of fibers affected in the corpus callosum. There is very little documentation of Rogers syndrome, and there are no documented cases of co-occurring Rogers syndrome in a patient with moyamoya disease. This report emphasizes the importance of documenting rare syndromic co-occurrences to expand the clinical understanding and improve recognition of atypical presentations in rare diseases.

## Case presentation

A 22-year-old female with a medical history of type 1 diabetes mellitus since childhood, Rogers syndrome, sensorineural hearing loss with a left cochlear implant, and moyamoya disease presented to the emergency department with altered mental status, left leg weakness, and delayed speech. Her symptoms had started three days before admission and progressed to urinary incontinence and reduced responsiveness, prompting her mother to seek emergency care. She had experienced an ischemic stroke involving bilateral frontal lobes five months prior, and was in the process of recovery with physical therapy. At her new baseline, she ambulated with a cane and exhibited mild left hemiparesis. Two weeks before the current presentation, her long-acting insulin dosage had been reduced from 25 to 13 units twice daily.

**Figure 1:**
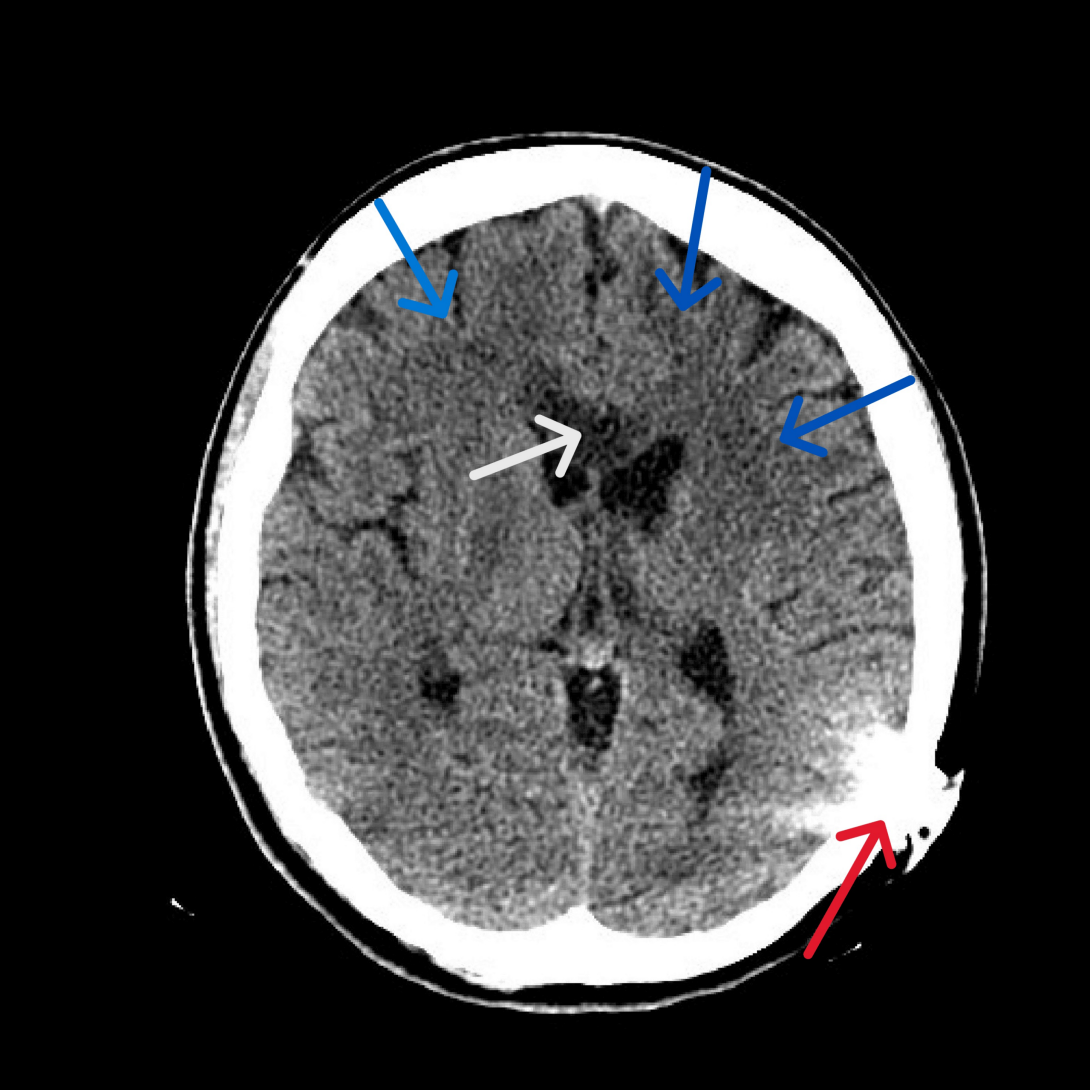
Initial brain CT The image illustrates hypoattenuation of the entire genu and anterior body of the corpus callosum (white arrow) with surrounding edema. The image also shows bilateral frontal infarcts with gliosis (blue arrows). The left cochlear implant artifact is also seen (red arrow) CT: computed tomography

On initial evaluation, she was tachycardic with a glucose of 419 mg/dL, bicarbonate of 9.6 mmol/L, and arterial blood gas showing pH of 7.274 and an anion gap of 16, consistent with diabetic ketoacidosis (Table 1). A non-contrast CT of the brain showed new hypoattenuation and edema involving the entire genu and anterior body of the corpus callosum, raising concern for acute or subacute infarction (Figure 1). The prior established bilateral frontal infarcts with gliosis were also noted. Given her clinical status and history of moyamoya, she was transferred to a tertiary center for further evaluation and neurosurgical evaluation and possible intervention. 

**Table 1:** Initial lab results pCO_2_: partial pressure of carbon dioxide; pO_2_: partial pressure of oxygen; HCO_3_: bicarbonate; WBC: white blood cell count; PT: prothrombin time; INR: international normalized ratio; PTT: partial thromboplastin time; BUN: blood urea nitrogen

Test	Result	Reference range
Sodium (arterial), mmol/L	136.6	135–145
Chloride (arterial), mmol/L	103	98–106
Potassium (arterial), mmol/L	3.94 mmol/L	3.5–5.0
Glucose (arterial), mg/dL	404 mg/dL	70–99
Calcium, ionized, mmol/L	1.28 mmol/L	1.12–1.32
Lactic acid, mmol/L	1.24 mmol/L	0.5–2.2
pH (arterial)	7.274	7.35–7.45
pCO_2_ (arterial), mmHg	21.1	35–45
pO_2_ (arterial), mmHg	126.3	75–100
HCO_3_ (arterial), mmol/L	9.6	22–28
WBC, K/uL	11.42	4.0–11.0
Hemoglobin, g/dL	15	12.0–16.0
Hematocrit, %	46.2	36–46
Platelet count, K/uL	323	150–450
PT, seconds	14.4	11.0–13.5
INR	1.1	0.8–1.2
PTT, seconds	30.3	25.0–35.0
Anion gap, mmol/L	16	8–16
CO_2_ (venous), mmol/L	9	22–29
Glucose (venous), mg/dL	350	70–99
Creatinine, mg/dL	0.64	0.6–1.3
BUN, mg/dL	14	7–20
Calcium (total), mg/dL	8.8	8.5–10.5
Phosphorus, mg/dL	4.2	2.5–4.5
Total protein, g/dL	7.7	6.0–8.3
Albumin, g/dL	3.7	3.5–5.5
Globulin	4.0	2.0–3.5

**Figure 2:**
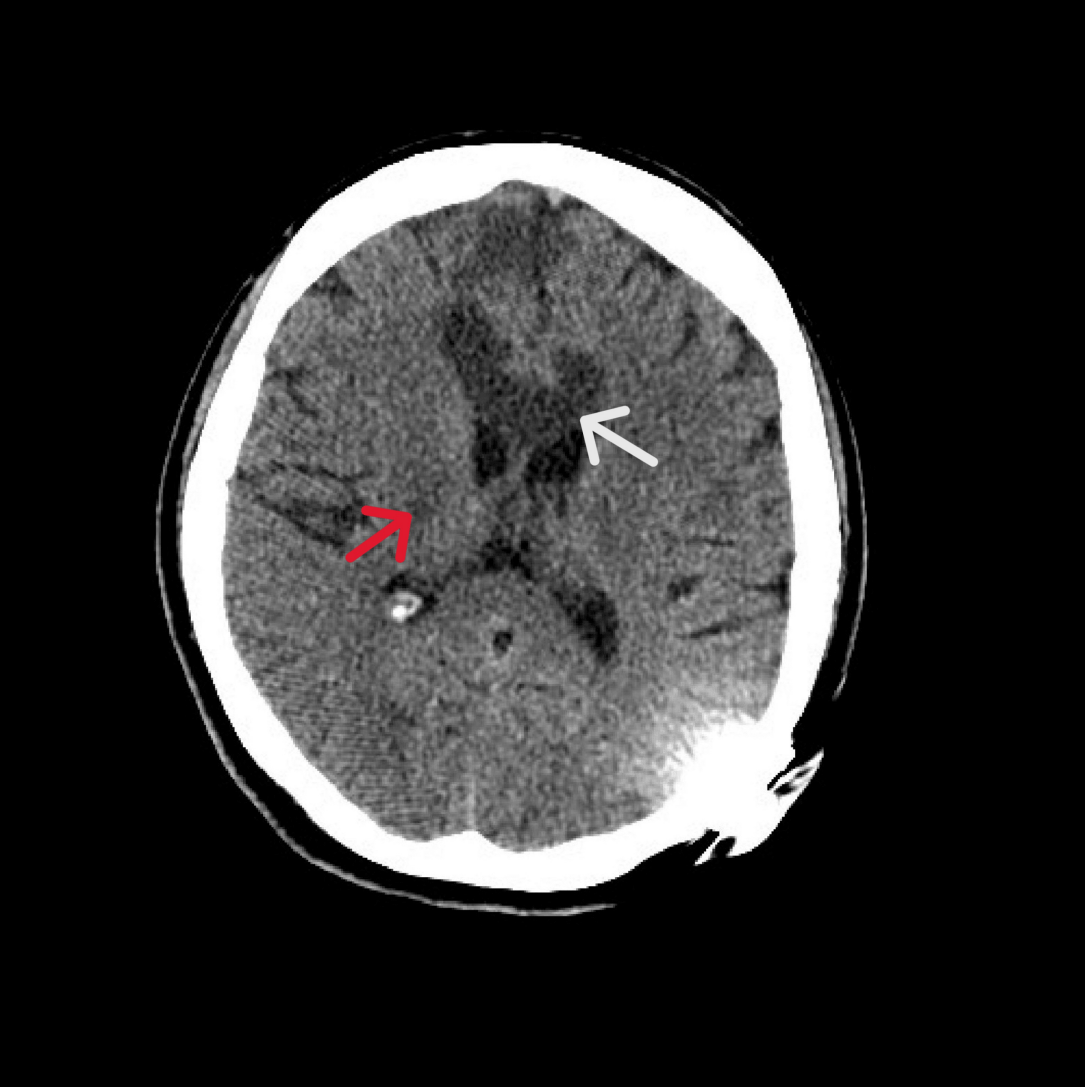
Repeat CT The image shows progressing infarcts within the corpus callosum (white arrow) and bilateral watershed territories (red arrow) CT: computed tomography

**Figure 3:**
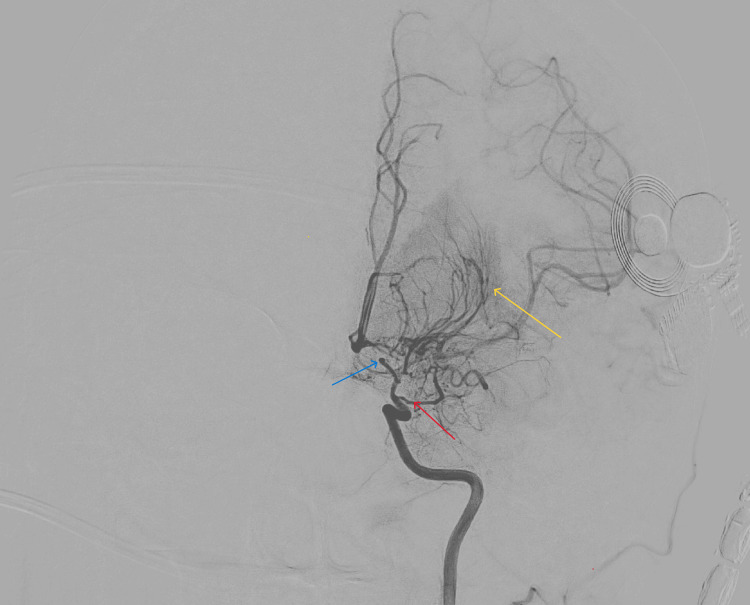
Cerebral angiography Cerebral angiography reveals progression of steno-occlusive disease involving the supraclinoid internal carotid artery, M1 (blue arrow), and A1 (red arrow) segments, with prominent leptomeningeal collaterals and the classic puff of smoke M1: first (horizontal) segment of the middle cerebral artery; A1: first (proximal) segment of the anterior cerebral artery

**Figure 4:**
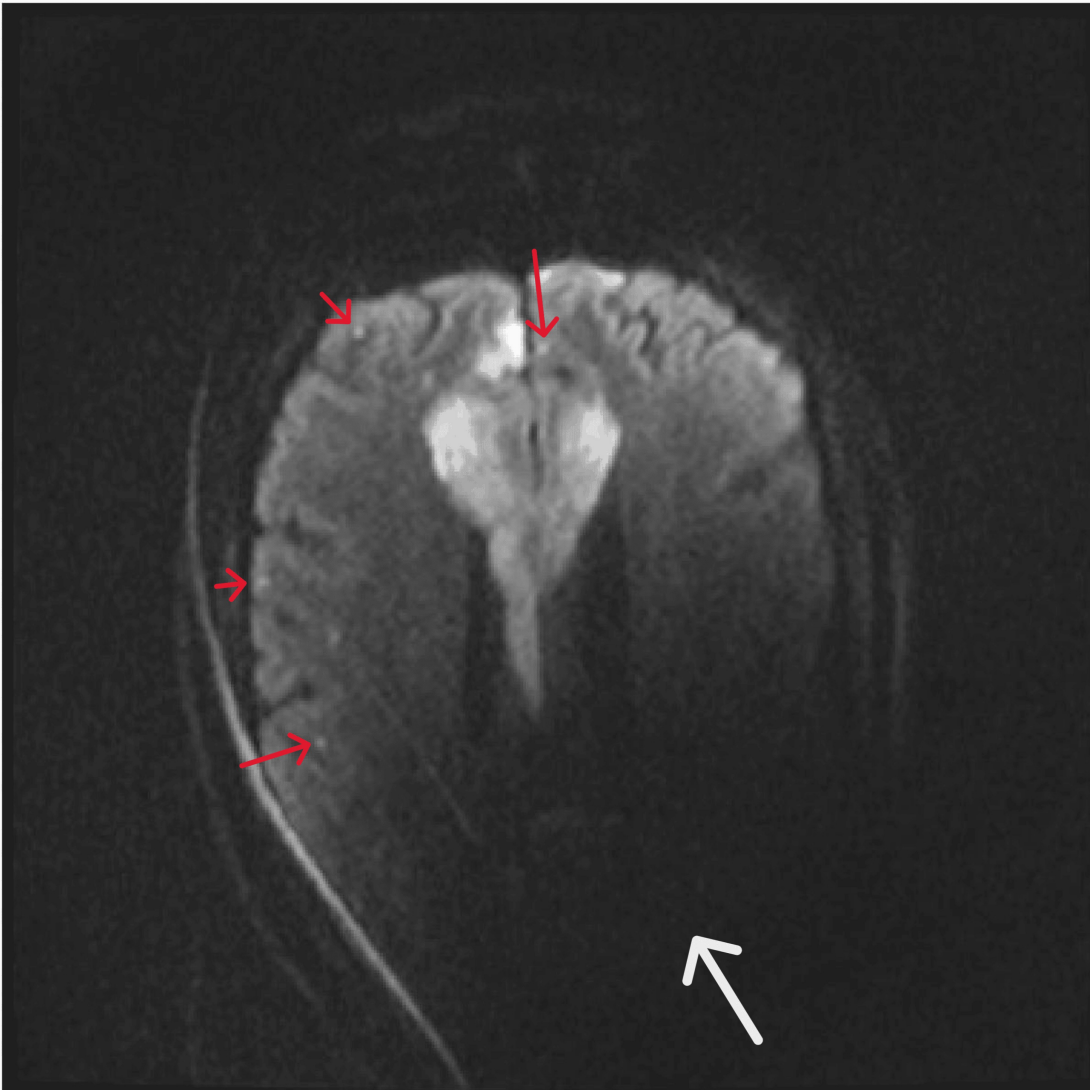
Brain MRI The image reveals acute multifocal punctate cortical infarcts (red arrows)scattered throughout bilateral frontal lobes and ACA-MCA distributions. Imaging is limited by the left cochlear implant artifact (white arrow) MRI: magnetic resonance imaging; ACA: anterior cerebral artery; MCA: middle cerebral artery

At the tertiary center, the patient remained encephalopathic with persistent left-sided weakness and urinary incontinence. Neurologic examination showed pronator drift of the left upper extremity and chronic leg weakness. She was following commands but aphasic. MRI of the brain revealed nondiagnostic diffusion-weighted imaging secondary to the patient’s cochlear implant, preventing detection of acute infarcts. However, new edema within the genu of the corpus callosum was noted, and an acute infarct could not be ruled out. Repeat CT confirmed evolving infarcts in the corpus callosum and bilateral watershed territories (Figure 2). Cerebral angiography revealed progression of steno-occlusive disease involving the bilateral supraclinoid internal carotid arteries, M1, and A1 segments, with prominent leptomeningeal collaterals; consistent with advanced moyamoya syndrome (Figure 3). Repeat MRI revealed acute multifocal punctate cortical infarcts scattered throughout bilateral frontal lobes and anterior cerebral artery-middle cerebral artery (ACA-MCA) distributions (Figure 4).

During this admission, the patient underwent a right frontal craniotomy for direct superior temporal artery-MCA (STA-MCA) bypass, a surgical procedure in which a branch of the external carotid artery (STA) is anastomosed to a cortical branch of the MCA to restore blood flow [9]. She also underwent bilateral parasagittal craniotomies for indirect revascularization, a technique that promotes new vessel formation by laying vascularized tissue over the ischemic cortex [10]. These procedures were already planned by the neurosurgical team with plans to perform at a later date due to decreased perfusion to the left hemisphere seen on Diamox perfusion scan; however, due to the clinical status of the patient and acuity, the process was accelerated.

**Figure 5:**
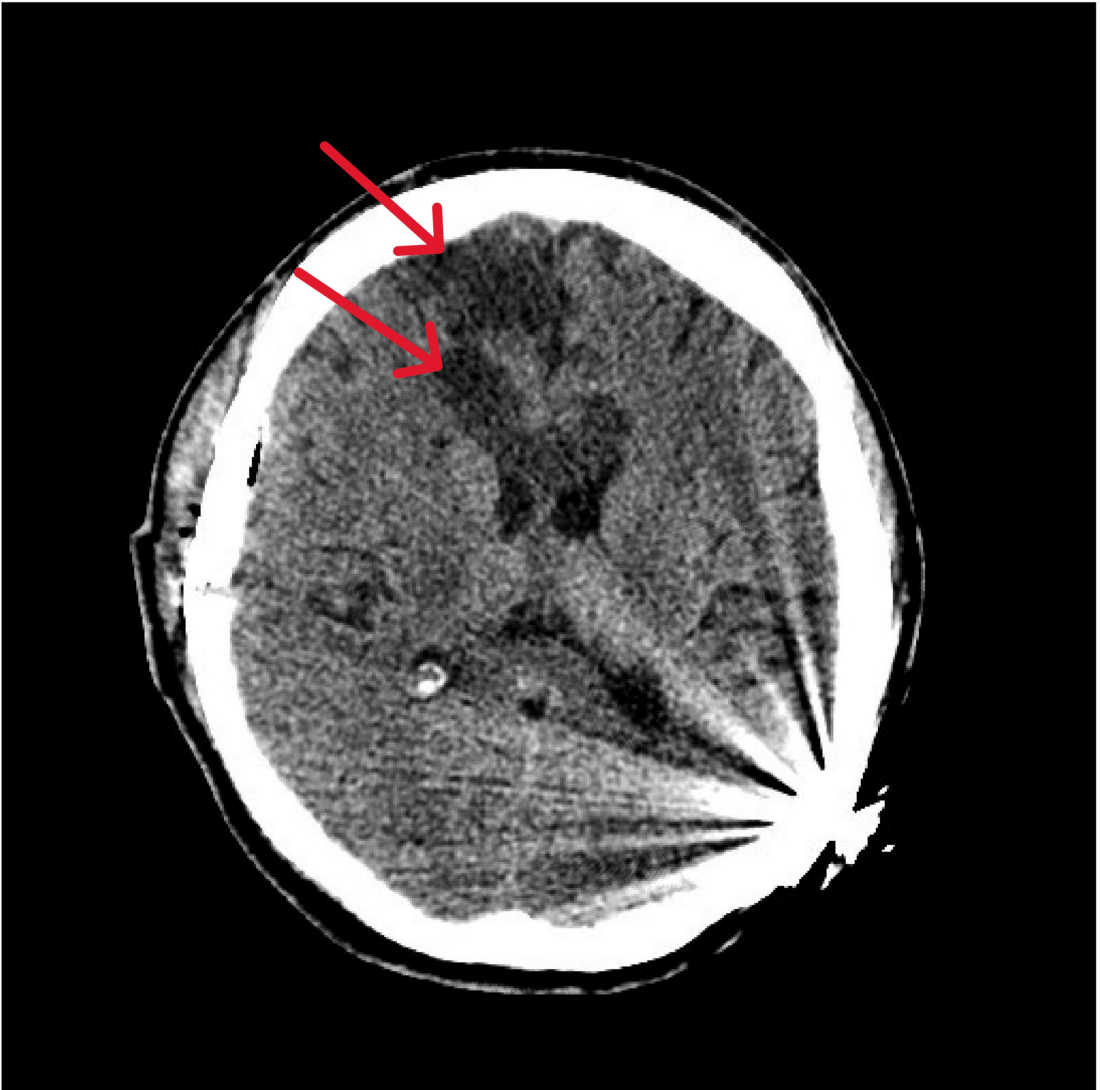
Follow-up postoperative CT imaging The image shows evolving infarction (red arrow) of the ACA and MCA territories without hemorrhagic conversion CT: computed tomography; ACA: anterior cerebral artery; MCA: middle cerebral artery

Follow-up CT imaging postoperatively showed evolving infarction of the ACA and MCA territories without hemorrhagic conversion (Figure 5). Video electroencephalogram revealed moderate-to-severe encephalopathy without epileptiform activity. A follow-up MRI seven days postoperatively demonstrated status post bilateral frontal and right temporal craniotomy with worsening bilateral ACA territories and cortical infarct, and a new right MCA territory small cortical infarct. Despite these new findings, the patient was not worsening clinically, and there were no surgical complications. 

Following surgery, the patient was transferred to an inpatient rehabilitation facility, where she required moderate assistance for mobility and was able to ambulate 50 feet using a gait belt. Neurological examination revealed 4/5 strength in the left hemibody, left-hand apraxia, and impaired coordination in the left upper extremity, consistent with callosal disconnection syndrome. She remained alert and cooperative with intact command-following but continued to experience generalized fatigue, persistent left-sided weakness, and impaired dexterity. Her recovery was gradual, and she continued to require ongoing multidisciplinary rehabilitation.

## Discussion

This report describes the first documented co-occurrence of Rogers syndrome and moyamoya disease in the medical literature, complicated by infarction of the corpus callosum resulting in a rare callosal disconnection syndrome. These rare pathologies led to an atypical stroke presentation and emphasize the complexity of vasculopathies, the importance of thorough workup, and the benefits of surgical intervention. Rogers syndrome (TRMA) is caused by mutations in the SLC19A2 gene, leading to defective thiamine transport and a clinical triad of DM, sensorineural hearing loss, and megaloblastic anemia. Diagnosis is typically clinical and confirmed through genetic testing [1,2,3]. Neurological manifestations in TRMA are poorly characterized but may include developmental delays and rare reports of unexplained strokes [11]. The pathophysiologic relationship between defective cellular metabolism and vascular integrity in this syndrome remains underexplored. 

Moyamoya disease is a progressive steno-occlusive arteriopathy affecting the intracranial internal carotid arteries and their proximal branches. It can present with ischemic or hemorrhagic strokes, often in the watershed areas of the anterior circulation [4,5]. Conventional angiography remains the gold standard for diagnosis, showing characteristic collateral vessel formation at the base of the brain [6]. Revascularization surgery, whether direct, indirect, or combined, is the mainstay of treatment and has been shown to reduce recurrent stroke risk [4,8].

Infarction of the corpus callosum is rare due to its robust dual blood supply from both the ACA and the posterior cerebral artery (PCA) territories. However, in the setting of extensive bilateral ACA disease and poor collateral flow, the callosal fibers become vulnerable to ischemia. The most affected areas are the genu and anterior body callosal fibers because of their predominantly ACA blood supply. Damage to these fibers results in callosal disconnection syndrome, characterized by impaired interhemispheric communication. Clinical features may include left-hand apraxia, agraphia, tactile anomia, and intermanual conflict. In our patient, MRI confirmed callosal infarction, and clinical signs such as left-hand dysfunction and bimanual coordination deficits were consistent with this syndrome. The metabolic insult of diabetic ketoacidosis may have contributed to the vulnerability of cerebral tissues that were already underperfused due to the underlying moyamoya arteriopathy. Furthermore, the impact of thiamine deficiency on vascular endothelial health may be synergistic in this case; however, this remains speculative.

This report emphasizes the importance of recognizing and exploring syndromic associations in young stroke patients, particularly when recurrent strokes occur. Careful evaluation is warranted for presentations involving atypical clinical or imaging findings, especially in the presence of metabolic or hematologic abnormalities. Multidisciplinary collaboration involving neurology, neurosurgery, endocrinology, genetics, and rehabilitation specialists is essential for accurate diagnosis and effective management.

## Conclusions

This report highlights the rare co-occurrence of Rogers syndrome and moyamoya disease in a young adult, complicated by infarction of the corpus callosum and resulting in callosal disconnection syndrome. It demonstrates how overlapping genetic, metabolic, and vascular pathologies can manifest in complex and atypical neurological presentations. Recognition of such rare syndromic associations is essential to guide timely diagnosis, appropriate surgical intervention, and coordinated rehabilitative care. Further research is needed to explore the potential pathophysiologic interactions between TRMA and cerebrovascular diseases like moyamoya.
